# Focal Segmental Glomerulosclerosis and Hyalinosis in a Patient with Spondyloarthritis: A Rare Renal Involvement Case Report

**DOI:** 10.31138/mjr.34.2.257

**Published:** 2023-06-30

**Authors:** Soumaya Boussaid, Houssem Tbini, Sonia Rekik, Ikram Mami, Lilia Ben Fatma, Samia Jammali, Hela Bargaoui, Hela Sahli, Lamia Rais, Mohamed Karim Zouaghi, Mohamed Elleuch

**Affiliations:** 1 Rheumatology Department, Rabta Hospital, Tunis, Tunisia,; 2 Nephrology, Dialysis, and Renal Transplantation Department, Rabta Hospital, Tunis, Tunisia,; 3 Faculty of Medicine of Tunis, University Tunis El Manar, Tunis, Tunisia

**Keywords:** case report, focal segmental glomerulosclerosis and hyalinosis, renal involvement, spondyloarthritis

## Abstract

**Background:**

During its course, spondyloarthritis (SpA) may be associated with extra-articular manifestations affecting several organs. Renal involvement is one of the most common extra-articular manifestations and is dominated by secondary amyloidosis (AA), immunoglobulin A (IgA) nephropathy, and urolithiasis. Other nephropathies such as Focal segmental glomerulosclerosis and hyalinosis (FSGS) are less common and are limited to few case reports.

**Case:**

We report the case of a patient followed for axial SpA, who consulted, after being lost to follow-up for 3 years, for elevated blood pressure and edema of both lower limbs associated with an hydrocele and bilateral pleural effusion. Biological examinations showed hypoproteinemia, hypoalbuminemia, and proteinuria. In this context of nephrotic syndrome, the diagnosis of FSGS was confirmed by renal biopsy. Furthermore, the etiological investigation ruled out the causes of secondary FSGS.

**Conclusion:**

Renal involvement is a sign of severity in SpA. Its detection and management should be part of the overall management of SpA.

## INTRODUCTION

Spondyloarthritis (SpA) is a chronic inflammatory rheumatism of the young adult male, and its prevalence is variable between 0.3 and 2% of the general population.^1^ It is expressed by axial or peripheral (articular and enthesitic) involvement. In addition, extra-articular manifestations may coexist and be life-threatening.^2,3^ Renal involvement is one of the most common extra-articular manifestations in SpA and accounts for 5–35% in SpA patients.^4^ Secondary amyloidosis (AA), immunoglobulin A (IgA) nephropathy, and urolithiasis are the most common causes of SpA-associated renal involvement in the Caucasian population.^5^ However, less common nephropathies such as focal segmental glomerulosclerosis and hyalinosis (FSGS) can be seen in SpA and are limited to rare case reports.^6,7^ We report a case of FSGS renal involvement diagnosed in a patient followed for SpA.

## CASE DESCRIPTION

This is the case of a 49-year-old man, a smoker at 20 pack-years, with no medical history except retrobulbar optic neuritis and an appendectomy. A family history of elevated blood pressure was reported in his father.

The patient was diagnosed with axial SpA in 2017. The diagnosis was made according to Assessment in Spondyloarthritis International Society (ASAS) 2009 criteria:^8^ inflammatory back pain for more than a year, age of onset<45 years, bilateral grade II sacroiliitis on radiographs, and elevated positive C-Reactive Protein (CRP) at 164mg/l [normal value (NV)≤8 mg/l]. No extra-articular manifestations were identified at the time of diagnosis. Blood pressure and blood sugar levels were normal. Non-Steroidal Anti-Inflammatory Drugs (NSAIDs) treatment (diclofenac and indomethacin) was initiated for the first two months after diagnosis. This treatment was quickly stopped upon the discovery of proteinuria at 0.3 g/24h. A corticosteroid therapy was therefore prescribed (10 mg of Prednisone) and we decided to start a biologic drug. Unfortunately, the patient was lost to follow-up from 2017 to 2020. During this time, he was self-medicated with NSAIDs and analgesics.

He consulted in December 2020 for white, soft, and pitting edema of the lower extremities. On examination, he weighed 78 kilograms, his body mass index was 24.6 kg/m^2^. His blood pressure was elevated at 180–200 mmHg for systolic and 100–120 mmHg for diastolic. Cardiac examination was normal. There was no hepatomegaly or pathological jugular turgor. Pulmonary examination found decreased breath sounds on pulmonary auscultation, which were absent at the base. Oxygen saturation was 97%. The abdomen was distended but painless to palpation. Bilateral flank dullness was noted on percussion. Examination of the external genitalia revealed a bilateral hydrocele. Osteoarticular examination revealed stiffness of the back without mobility limitation, and mobility limitation of the hips. There were no other joint involvements or abnormalities on general examination.

Laboratory examinations had shown: CRP at 51 mg/l [NV≤8 mg/l], creatinine and estimated glomerular filtration rate (eGFR) at 53.04 μmol/l [NV: 55–100 μmol/l] and 151 ml/min [NV: 90–130 ml/min] respectively, 24-hour proteinuria at 5.9 g/24h [NV<30 mg/24h], total serum protein at 46 g/l [NV: 60–80 g/l], and serum albumin at 14.4 g/l [NV: 35–50 g/l]. Otherwise, there was no hematuria. These clinical and biological data led to the diagnosis of impure nephrotic syndrome with hypertension. Furthermore, the SpA was highly active, according to the disease activity scores (BASDAI: Bath Ankylosing Spondylitis Disease Activity and ASDAS-CRP: Ankylosing Spondylitis Disease Activity Score) which were 6.3 and 4.95, respectively.

**Figure 1.**
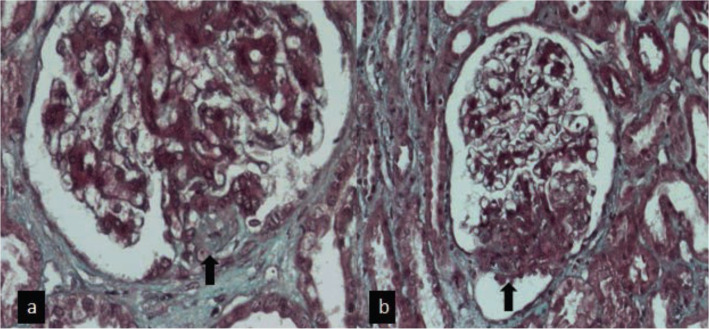
**(A)** Glomerulus in a renal biopsy image with focal segmental glomerulosclerosis lesion (arrow). Light microscopy (Trichrome x 400). **(B)** Glomerulus in a renal biopsy image with focal segmental glomerulosclerosis lesion (arrow). Light microscopy (Trichrome x 200).

In order to explore the nephrotic syndrome; we performed a renal biopsy whose histologic examination concluded with an FSGS involvement. In fact, the biopsy was performed under ultrasound guidance and brought 3 cortical fragments with 25 glomeruli, none of which were sclerotic. In light microscopy, segmental and focal hyalinosis lesions were observed in 5 glomeruli per section plane. Podocytes had a turgid appearance with abundant cytoplasm. The walls of the glomerular capillaries were normal in appearance, no visible deposits were observed, and Cango Red staining was negative. Moreover, a non-inflammatory interstitial fibrosis and tubular atrophy occupying 20% of the examined parenchyma was observed. As for the vessels, there were 2 to 3 interlobular arteries of normal appearance. The arteriolar sections showed moderate hyalinosis (**Figure 1**).

For immunofluorescence, hematoxylin and eosin staining was performed in 7 glomeruli and revealed membrane staining with IgA antisera, segmental glomerular IgM deposition and negative fibrin, C1q, C3, and IgG antibodies.

Only light microscopy and immunofluorescence analyses were performed, as an electron microscope was not available.

The patient was hospitalised and an etiological investigation has been initiated. We ruled out viral infections (human immunodeficiency virus, hepatitis B virus, parvovirus B19, and cytomegalovirus) by negative viral serology, drug and toxic causes by interrogation, and causes of FSGS by glomerular hyperfiltration by normal renal ultrasound and urological exploration and given the absence of diabetes, obesity, sickle cell disease and cyanotic cardiac disease. At the same time, we started corticosteroid therapy (Prednisone) at a dose of 1 mg/kg/day in association with furosemide (initially intravenously at a dose of 200 mg then a gradual decrease to 40 mg per OS), spironolactone (100 mg per OS per day), and ramipril (2.5 mg per OS per day). In addition, five albumin infusions were prescribed.

The patient was initially monitored daily (during his stay in hospital). Then he was examined as an outpatient every week for the first month and then every 2 weeks. Proteinuria was assessed at each visit by urine dipstick, and a 24-hour proteinuria determination was performed every 4 weeks. Blood analysis (serum protein, albumin, and creatinine) was also performed.

After 12 weeks of treatment, the patient lost 15 kg of weight. His creatinine and eGFR levels were 51.33 μmol/l and 156 ml/min, respectively. His total serum protein and albumin levels were 64 g/L and 32.4 g/L, respectively. However, he still had an estimated proteinuria of 2.3 g/24h (3 crosses on the urine dipstick).

Concerning SpA management, the patient is currently waiting for an agreement for the coverage of a biologic drug (Adalimumab) from the social insurance funds. He is only receiving analgesic treatment.

## DISCUSSION

We report a case of FSGS occurring in a patient with axial SpA. This involvement is extremely rare, and this is the fifth case reported in the literature to the best of the authors’ knowledge. The renal involvement responded well and improved after appropriate management. However, we were not able to assess the therapeutic response to biologics since we have not yet received approval from the social insurance funds for treatment.

During SpA, renal involvement can be expressed by different symptoms: edema, proteinuria, hematuria, arterial hypertension, urolithiasis, renal failure and nephrotic syndrome.^5–7^ This impairment generally reflects a long-standing disease (with an average of 6.6 to 19.4 years),^6^ and has clearly decreased since the arrival of biologic drugs.^7,9^ AA amyloidosis, IgA nephropathy, and urolithiasis are the most common causes of renal involvement in SpA in the Caucasian population.^6,10^

The prevalence of these etiologies varies according to the studies and more than 90% of renal damage is explained only by amyloidosis (62%) and IgA nephropathy (30%).^11,12^ In contrast, other etiologies are estimated at less than 8% and are limited to case reports. Cases of membranous glomerulonephritis, mesangial glomerulonephritis with C3 and IgM deposits, and membranoproliferative glomerulonephritis have been reported.^7,10,13,14^

Extremely rare cases of SpA-FSGS association have also been reported, including 3 cases in 3 Tunisian series.^6,15–18^ Interestingly, many reported cases were from Tunisia. A genetic predisposition or an environmental effect may explain this association otherwise. However, in these cases, the association between SpA and FSGS has not been clearly demonstrated.

Causes of secondary FSGS are various.^19^ We have excluded in our patient viral and drug/toxic causes. We have also ruled out the hypothesis of secondary FSGS by glomerular hyperfiltration despite an eGFR of 151 ml/min after eliminating the causes that can induce it, ie, a reduced nephron mass and adaptive response (by ultrasound and urological exploration and by ruling out diabetes, obesity, sickle cell disease and cyanotic cardiac disease). Furthermore, elevated blood pressure does not seem to be a cause of FSGS in our case. In fact, our patient had a normal blood pressure when proteinuria first appeared. As for NSAIDs, our patient was taking NSAIDs for a long time, and association NSAIDs-FSGS may be possible. Lastly, an idiopathic form of FSGS would also remain possible.

A review of the literature showed that NSAIDs-induced renal damages are mainly acute interstitial nephritis, acute tubular injury, minimal change disease, papillary necrosis, and rarely membrane nephropathy.^17,20^ Data in the literature about the relationship between NSAIDs and FSGS are limited; an association has been reported by some authors, even for topical NSAIDs.^21,22^ These data could support arguing that our patient’s FSGS may be related to SpA. However, Markowitz et al. and Paueksakon et al., in recent studies, state that the available evidence does not support a relationship between NSAIDs and FSGS.^20,23^

In summary, there are no studies, as well as epidemiologic evidence, that directly demonstrate the causal relationship between SpA and FSGS. Moreover, the role of NSAIDs, which are the main treatment for SpA, as a cause of FSGS remains unclear.

Our patient will be put on Adalimumab. The latter has been used since 2006 for the treatment of SpA.^24^ It has also been used in refractory cases of FSGS since 2014, it reduces proteinuria and protects kidney function from degradation, it has also been reported as an alternative in cases of resistance or allergy to Rituximab in FSGS.^25,26^

## CONCLUSION

We report a rare case of FSGS in a patient with SpA. Screening for renal involvement should be part of the follow-up strategy for SpA. Renal biopsy is an important tool in these cases; it has both diagnostic, therapeutic and prognostic value. Rare etiologies such as FSGS may exist in SpA; and a secondary cause of FSGS should be sought systematically.

## PATIENT PERSPECTIVE

The patient reported great improvement with the treatment received for the kidney damage. However, he is currently experiencing a flare-up of his SpA (with significant back pain).

## Data Availability

The datasets used and/or analysed during the current study are available from the corresponding author on reasonable request.

## LIST OF ABBREVIATIONS:

AA: Secondary amyloidosis
ASAS: Assessment in Spondyloarthritis International Society
ASDAS: Ankylosing Spondylitis Disease Activity Score
BASDAI: Bath Ankylosing Spondylitis Disease Activity
CRP: C-Reactive Protein
eGFR: estimated glomerular filtration rate
FSGS: Focal segmental glomerulosclerosis and hyalinosis
Ig: Immunoglobulin
NSAIDs: Non-Steroidal Anti-Inflammatory Drugs
NV: Normal value
SpA: Spondyloarthritis
